# Catecholamine Contents of Different Region of Adult Rat Brain Are Altered Following Short and Long-term Exposures to Pb^+2^

**Published:** 2013

**Authors:** Minoo Moshtaghie, Pedram Malekpouri, Mohammad Saeed-zadeh, Manuchehr Messripour, Ali Asghar Moshtaghie

**Affiliations:** a *Department of Environment and Energy, Science and Research Branch, Islamic Azad University, T ehran, Iran.*; b *Young Researchers and Elites Club, Science and Research Branch, Islamic Azad University, Tehran, Iran.*; c *Department of Clinical Biochemistry, School of Pharmacy, Isfahan University of Medical Science, Isfahan, Iran.*; d *Department of Biochemistry, School of Basic Science, Falavarjan Branch, Islamic Azad University, Isfahan, Iran.*

**Keywords:** Lead, Catecholamine, Brain region, Pb^+2^

## Abstract

Catecholamine is a group of neurotransmitters that is believed to be responsible for the normal function of animal brain. Physiological and behavioral changes of human body have been reported due to the damage of the brain function following lead exposure. Due to the assumption of lead disposal in brain tissue with two year for its half-life, which results in alteration of brain function, we investigated the ability of lead to change the brain catecholamines during short and long-term studies. Rats were exposed daily with varying amounts of lead and catecholamine contents of cerebellum, mid-brain and brain cortex were determined. Acute peritoneal administration of single dose of lead as lead acetate (260 μmol/Kg) after 2 h reduced (p *< *0.05) the catecholamine levels of cerebellum, mid-brain and cortex part by 34.9%, 35.44% and 23.8%, respectively. The extension of experiment time to 5 h, significant (p < 0.05) reductions in catecholamine levels of mentioned regions of brain by 32.35%, 12.35% and 19.3% were seen respectively. Daily intraperitoneal administration of 10 μmol/Kg lead for 30 and 60 days reduced catecholamines levels of cerebellum (22.22% and 30.44%), midbrain (12.48% and 26.27%) and brain cortex (11.58% and 26.7%) respectively. It might be concluded that brain dysfunction in lead intoxicated rat occurred through the reduction in the catecholamine levels of different parts of brain. Lead might be therefore considered as a probable factor in causing neurological disease in lead exposed man.

## Introduction

The increasing lead (Pb^+2^) emission in the environment is bringing lots of health problems in human throughout the word. Utilization of this metal in industrial activity, smelting, paints, ceramic dish and *etc. *are concerned as major sources for Pb^+2^ disposal in our environment (1). Exposure of human to Pb^+2^ could be occurred through gastrointestinal (water and food contaminations) and respiratory (air-polluted) systems, and then absorbed and stored in tissues including bone, brain, *etc*. (2). The effects of Pb^+2^ on human health lead to the appearance of anemia, bone disease, renal dysfunction, liver hyperplasia, encephalopathy and many kinds of cancer (2-8).

 Since the half-life of Pb^+2^ in brain is about two years, the toxicity of Pb^+2^ on Central Nervous System (CNS) is more common among the children and adult and might provide some behavioral disorders, learning and hearing disabilities and cognition impairments (9). The oxidative damage of Pb^+2^ on brain biomolecules including DNA, lipids and proteins have been reported and named as one of the most important mechanisms for Pb^+2^ toxicity (1, 10, 11). Furthermore, Pb^+2^ is able to substitute calcium in calmodulin and alter its function as essential nutrient for signal transmission and neurotransmission (12, 13). The inhibitory effect of Pb^+2 ^on Na^+^/K^+^-ATPase activity by focusing on its role for linking the extracellular to intracellular signals at neuron level has been evidenced by NourEddine *et al*. (14). 

Although encephalopathy is more common especially in children, severe complication of Pb^+2^ poisoning resulted in encephalopathy and it is associated with ataxia which could be due to the exposure of Pb^+2^ in particularly workplace (15). The pathogenesis of Pb^+2^ encephalopathy in the cerebellum region of brain could be linked to the more susceptibility of cerebellum capillary to Pb^+2^ than other regions. It however well evidenced that brain Pb^+2^ retention is prolonged and unaffected by some kind of chelating therapy (16). Several investigations have shown that astroglial cells accumulate and store Pb^+2^ in intracellular (17) suggesting that these cells may act as a storehouse for Pb^+2^ in CNS. Other observations show alterations in motor coordination and cortical function and make damage to cholinergic and neurotransmission system. Cholinergic function requires adequate amount of neurotransmitters such as catecholamine, acetylcholine and *etc*. (18).

Catecholamines represent a group of neurotransmitters including dopamine, adrenaline and noradrenalin. They are present in CNS and fulfill a variety of functions such as motor control, cognition, emotion, memory processing, and endocrine regulation in human and animal bodies (19). The main sites of catecholamine production are brain, chromaffin cells and the sympathetic neurons. It has been clearly demonstrated that the most important neurotransmitters in the central nervous system are dopamine and noradrenalin (20). Catecholamines play an important role in the control and regulation of numerous brain functions and they also involve in different neurodegenerative disorders like Alzheimer Disease (AD) and Parkinson Dementia (PD) (21). The role of catecholamines in health and disease have been described long time ago by variety of researchers. In this regard, changes in catecholamine levels are associated with some environmental stresses and measurement of this parameter is a useful tool for the recognition of related promoting agents (20). 

Previously, the devastator effects of toxic elements on catecholamine levels in different brain regions have been reported. In this respect, Yadav *et al*. (22) indicated that arsenic can decrease the level of catecholamine in rat brain. Furthermore, chromium leads to decrease in catecholamine level in rat brain (23). In another experiment, the lessening pattern for catecholamine following short and longterms aluminum exposure were observed by Moshtaghie *et al. *(24). Influences of other toxic metals like nickel and mercury are controversial and need more investigation. 

Due to the prevalence of Pb^+2^ production in industrial and developing countries, the exposure of this toxic element may influence the human health, particularly children health, which may retard and damage their normal growth and also affect their brain functions (14). Therefore, the major aim of the present investigation was to establish short and long-term effects of Pb^+2^ on the catecholamine levels as an important neurotransmitter for brain function in different parts of brain. Rats were used as an animal model for this project as well.

## Experimental

*Chemicals*

All chemicals were of reagent grade and highest purity available, and were obtained from Sigma Chemical Company (Sigma, Germany) to minimize the metal contamination. Deionized water was used throughout this project. All glasswares were soaked overnight in 10% HNO_3 _and were then washed three times with distilled water and then with deionized water. Plasticwares were also prewashed by 10 mmol EDTA followed by three washes with distilled water and finally deionized water.

*Treatment of animals*

Male Wistar rats were purchased from Pasteur Institute (Tehran, Iran) and maintained on animal house until achieving the desired weight. The animals’ weights were within the range of 200-250 g at the start of experiment. These animals supported ad libitum for food and water and also normal light and darkness (12 L: 12 D). The temperature of their environment remained steady during the experiment (20 ± 3°C). This study was carried out as three separated experiments including 2 and 5 h, and 30 and 60 days.

*Experiment 1 (short-term study)*

The animals were divided into two groups, with the first group serving as control and the other as experimental group. The experimental group was exposed to sub-lethal concentration of Pb^+2^ (260 μmol Kg-1 BW), lower than LD_50_ for intraperitoneal injection in rat, which is 150 mg Kg^-1^(25). Desired dose of Pb^+2^ ion was prepared by dissolving Pb^+2^ as acetate salt (Pb(CH_3_COO)_2_.3H_2_O) in normal saline (NaCl: 9 gL^-1^). The mentioned amount was injected intraperitoneally using syringe into rat abdomen and the animals were tested following 2 and 5 h of injection time. Control group received the same volume of normal saline (0.3 mL) as used for experimental animals.

*Experiment 2 (long-term study)*

For investigation of Pb^+2^ exposure on catecholamine levels of rat brain in long-term, a treatment with 10 μmolKg^-1^ was prepared and each individual (five) received the same amount of intraperitoneal Pb^+2^ daily for periods of 30 and 60 days. Control animals received only the same volume of normal saline daily. At the end of 30 and 60 days of administration, all animals were prepared for brain catecholamine measurement.

*Sample preparation*

At the end of each period of experiment, all treated rats (five) were killed by decapitation. Hereafter, the animal’s brain has been removed quickly by dissection of cranial cavity and washed by ice cold saline. The different regions of brain were isolated using method described by Glowinsky *et al. *(26) and the following tests were done.

*Catecholamines measurement*

The rat’s brain was applied for quantifying of catecholamine levels. In this regard, different regions of brain have been homogenized using perchloric acid (0.1 M) and EDTA 1% for 5 min. The homogenate was centrifuged for 20 min with 10000 rpm at 4°C. The supernate fluid was then separated and the containing catecholamines were extracted by using of alumina column. This procedure has been performed when the supernatant pH was reached 8.6. This elevation will be achieved by adding EDTA and sodium bisulfite (12.5%). After that, the supernatant was added to alumina (Al_2_O_3_) and allowed to adhere onto alumina for 15 min at the room temperature. The alumina was then washed twice with distilled water. For separation of catecholamines, HCl 1% was used finally (27). The levels of catecholamines were measured using spectrofluorimetric technique and reported as ng of catecholamines per mg protein in each brain region.

*Protein determination*

The amounts of tissue protein were determined according to the method of Lowry *et al. *(28).

*Statistical analysis*

The obtained data were subjected to statistical analysis using SPSS software (version 18). In all cases, the one-way analysis of variance (ANOVA) was used to compare the mean of each treatment with control group. The LSD complementary test was conducted to elucidate the exact differences at p-value lower than 0.05. Data are presented as mean ± SD (Standard Deviation) for all cases.

## Results and Discussion

**Figure 1:**
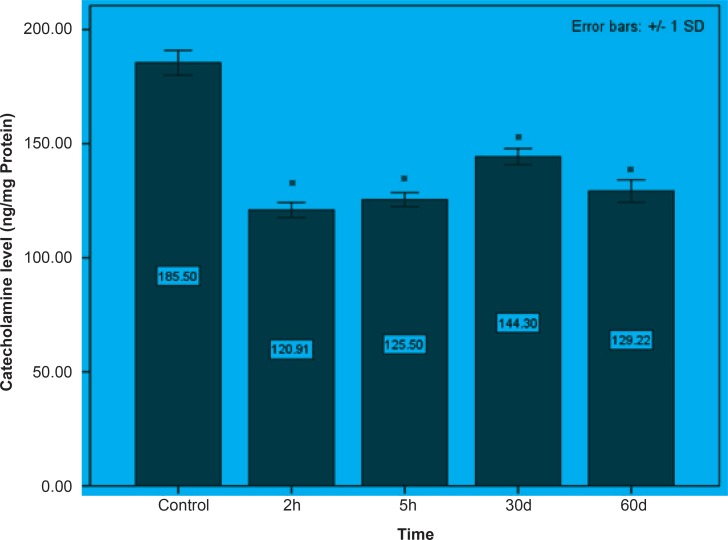
The effect of Pb^+2^ on catecholamine in cerebellar part of rat’s brain during the short and long periods. Each column is the mean of 5 observations. Asterisks indicate significant differences (p *< *0.05) between treatments and control

**Figure 2:**
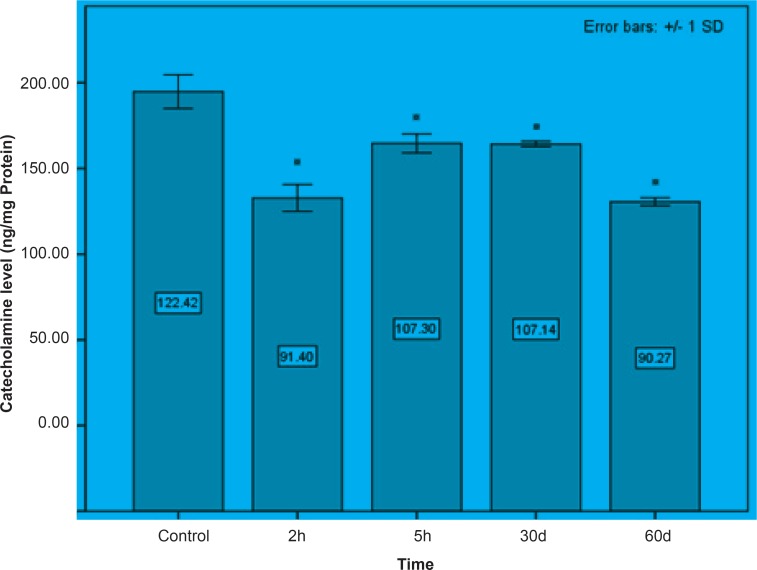
Mid-brain concentrations of catecholamine following the short and long periods of Pb^+2 ^injection. All data appears as mean ± SD (n = 5). Significances are expressed by * at p < 0.05 when compared with control treatment.

**Figure 3:**
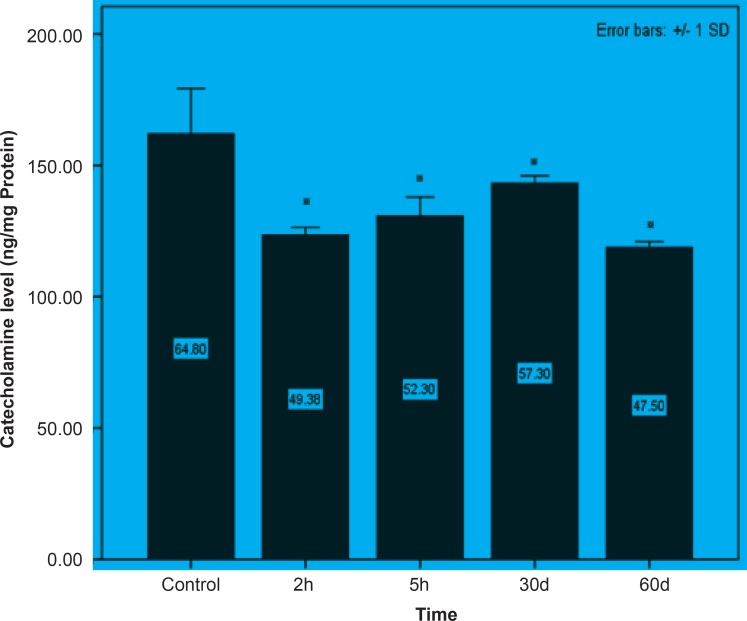
Short and long-term effects of Pb^+2^ on catecholamine in cortex part of rat brain. All data are shown as mean ± SD. Significant (p < 0.05) differences from control group are marked by asterisks

Initially, rats were exposed to Pb^+2^ (260 μmol Kg^-1^ BW) with a single dose and decapitate after 2 h. Reductions about 34.9%, 35.44% and 23.8% were observed in catecholamine levels of cerebellum, mid-brain and brain cortex respectively when compared to control. There were significant reductions in catecholamine levels of cerebellum (32.35%), mid-brain (12.35%) and cortex (19.3%) of rat administrated with the same dose of Pb^+2^ as previous group and decapitated after 5 h (Figures 1, 2 and 3).

 In the second stage of the project, the longterm effects of Pb^+2^ on the catecholamine content of different parts of rat brains was also investigated. For this purpose, rats were injected daily with (10 μmol Kg^-1^ BW) Pb^+2^ for 30 and 60 days. The catecholamine levels of cerebellum, mid-brain and cortex were reduced (p < 0.05) by 22.22%, 12.48% and 11.58% respectively following the daily injection of Pb^+2^ for 30 days. A new pronounced reduction of brain catecholamines following Pb^+2^ exposure for 60 days was observed in cerebellum by 30.44%, mid-brain by 26.27% and cortex by 26.7% in comparison with control group (Figures 1, 2 and 3). 

In the present study, we evaluated the effect of different treatments of Pb^+2^ on catecholamine level of adult rat brain. In all concentrations tested, significant (p < 0.05) reductions in brain catecholamine were observed. In agreement to our finding, Devi *et al*. (29) and Smith and Cass (30) suggested that Pb^+2^ can lead to decrease in catecholamines. Long-term memory and also learning function might be affected by Pb+2administration (14). In addition, all neurotransmitters were observed to be decreased following the different Pb^+2^ treatment (31). Previous researches indicated that Pb^+2^ treatments (even in low concentration) resulted in high level of serum Pb^+2^ and therefore its amount is correlated with accumulation of that in brain tissue (32). Intracellular accumulation of Pb^+2^ interacts with high molecular weight proteins in C6 glioma cells and lead to alter the membrane transport properties for copper (33) and therefore copper deficiency might impairs catecholamine levels of brain (34). The reduction of catecholamine concentrations in all brain parts was due to the deleterious effect of Pb^+2^ in one hand and declined stimulant effect of copper in the other hand, on production of Superoxide Dismutase (SOD) (11, 35). This enzyme can protect oxidizable compounds such as catecholamines against toxic effects of oxygen (36). Therefore, copper can likely provide more SOD to protect catecholamine in brain. Recently, Benetti *et al*. (37) showed that Cuprizone can provide some status like symptoms that we observe in neurodegenerative disorders. They suggested that it probably engender a chronic copper deficiency by cleating to this element. Furthermore, impairment of the activity of dopamine beta hydroxylase following copper deficit could likely impact on brain development. This process will be done through changing in brain norepinephrine (34). 

The probable mechanism for the effect of Pb^+2^ on the reduction of catecholamines might be related to the competition between Pb^+2^ with either magnesium and/or zinc which may lead to deficiency (ies) of these metals (38-40). These metals are necessary for the conversion of Dihydroneopterin triphosphate to Tetrahydrobiopterin. Tetrahydrobiopterin is an essential cofactor for tyrosine hydroxylase, tryptophan hydroxylase, phenylalanine hydroxylase, and nitric-oxide synthase. These enzymes synthesize neurotransmitters, *e.g.* catecholamines, serotonin, and nitric oxide (41). In overall, Pb^+2^ may likely impair the abovementioned process by reduction in essential elements and changing in the structure of Tetrahydrobiopterin. The reduction in catecholamines content may however be as a result of either decreasing in the catecholamines following Pb^+2^ accumulation in brain and/or might be related to the preventing role of Pb^+2^ to release this neurotransmitter (40). 

Pb^+2 ^may alter the aminergic system by decreasing the mitochondrial monoamine oxidase

and tyrosine hydroxylase activity. The ability of Pb^+2^ for the reduction of catecholaminergic transmission may be occurred either by inhibition of the synthesis of dopamine and its acceleration auto oxidation (29, 42) or by an inhibition of postsynaptic dopamine receptors (14, 43). The investigation showed that Pb^+2^ can block the activation of Ca^+2^/phosphide dependent protein kinase C (PKC). The PKC isoforms are known as signal transducers in CNS that play role in regulation of vesicles movement and secretion in synapse (44). 

Results from the present study showed that Pb^+2^ even in short time of exposure and also in the low level of concentration had the devastating effect on the brain catecholamine. Reductions in catecholamine were more perceivable in cerebellum and it may however assume that this part of brain is much more susceptible to Pb^+2^ toxicity. 

As indicated in all Figures (1, 2 and 3), animals regardless of tested region show more decrease after 2 h from the beginning of experiment and since then, there was a steady increase in catecholamine level of rat brain at the time of 5 h, but it does not return to the control level. Anyway, this can be interpreted as a compensatory response of the brain cell to the toxic effect of Pb^+2^ following short-term exposure. Nevertheless, this response was not observed following long-term Pb^+2^treatment and a simple reduction was shown. Additional experiment should be employed to examine the involved mechanisms, which are mentioned so far. Applying molecular technique can clarify the exact mechanism by which Pb^(+2)^ interferes with catecholamine metabolism.

## References

[1] Verstraeten SV, Aimo L, Oteiza PI (2008). Aluminium and lead: molecular mechanisms of brain toxicity. Arch. Toxicol.

[2] Mudipalli A (2007). Lead hepatotoxicity & potential health effects. Indian J. Med. Res.

[3] Payton M, Hu H, Sparrow D, Weiss ST (1994). Low-level lead exposure and renal function in the normative aging study. Am. J. Epidemiol.

[4] Angle CR, Thomas DJ, Swanson SA (Biometals). Osteotoxicity of cadmium and lead in HOS TE 85 and ROS 17/2.8 cells: Relation to metallothionein induction and mitochondrial binding.

[5] Nwankwo EA, Ummate I (2006). Environmental Lead Intoxication and Chronic Kidney Disease: A Review. Internet J. Nephrol.

[6] Hegazy AA, Zaher MM, Abdel hafez MA, Morsy AA, Saleh RA (2010). Relation between anemia and blood levels of lead, copper, zinc and iron among children. BMC Res. Notes.

[7] NematiKarimooy H, Balali Mood M, Hosseini M, Shadmanfar S (2010). Effects of occupational lead exposure on renal and nervous system of workers of traditional tile factories in Mashhad (northeast of Iran). Toxicol. Ind. Health.

[8] Sahu JK, Sharma S, Kamate M, Kumar A, Gulati S, Kabra M, Kalra V (2010). Lead encephalopathy in an infant mimicking a neurometabolic disorder. J. Child Neurol.

[9] Gulson BL, Pounds JG, Mushak P, Thomas BJ, Gray B, Korsch MJ (1999). Estimation of cumulative lead releases (lead ﬂux) from the maternal skeleton during pregnancy and lactation. J. Lab Clin. Med.

[10] Moreira EG, Rosa GJ, Barros SB, Vassilieff VS, Vassillieff I (2001). Antioxidant defense in rat brain regions after developmental lead exposure. Toxicology.

[11] Yun HJ, Kim I, Kwon S-H, Kang J-S, Om A-S (2011). Protective Effect of Chlorella vulgaris against Lead- Induced Oxidative Stress in Rat Brains. J. Health Sci.

[12] Vazquez A, Pena O (2004). Lead impairs long-term memory and blocks learning-induced increases in hippocampal protein kinase C activity. Toxicol. Applied Pharmacol.

[13] Reddy GR, Wari B, Devi C, Chellu S (2006). Developmental lead Neurotoxicity. Neurotoxicology.

[14] NourEddine D, Miloud S, Abdelkader A (2005). Effect of lead exposure on dopaminergic transmission in the rat brain. Toxicology.

[15] Mani J, Chaudhary N, Kanjalkar M, Shah P (1998). Cerebellar ataxia due to lead encephalopathy in an adult. J. Neurol. Neurosurg. Psychiatry.

[16] Goldstein GW, Asbury AK, Diamond I (1974). Pathogenesis of Lead Encephalopathy Uptake of Lead and Reaction of Brain Capillaries. Arch. Neurol.

[17] Opanashuk LA, Finkelstein JN (1995). Induction of newly synthesized proteins in astroglial cells exposed to lead. Toxicol. Appl. Pharmacol.

[18] Bressler JP, Goldstein GW (1991). Mechanisms of lead neurotoxicity. [Review]. Biochem. Pharmacol.

[19] Kobayashi K (2001). Role of Catecholamine Signaling in Brain and Nervous System Functions: New Insights from Mouse Molecular Genetic Study. J. Invest. Dermatol. Symp. Proc.

[20] Chattoraj SC, Watts NB, Tietz NW (1986). Endocrinology. Texbook of clinical chemistry.

[21] Cropley VL, Fujita M, Innis RB, Nathan PJ (2006). Molecular imaging of the dopaminergic system and its association with human cognition. Biol. Psychiatry.

[22] Yadav RS, Shukla RK, Sankhwar ML, Patel DK, Ansari RW, Pant AB, Islam F, Khanna VK (2010). Neuroprotectiveeffect of curcumin in arsenic-induced neurotoxicity in rats. Neurotoxicology.

[23] Moshtaghie AA, Afrang M, Mesripour M (2004). Changes in catecholamines and acetylcholinesterase levels of crebellum, mid-brain and brain cortex in chromium treated rats. Iranian J. Pharm. Res.

[24] Moshtaghie AA, Rahimi S, Messripour M (1996). Changes in catecholamine levels of cerebellum, mid-brain and brain-cortex in aluminiumintoxified rats. Indian J. Pharmacol.

[25] Lyn P (2006). a review of the literature. Part I: exposure, evaluation and treatment. Altern. Med. Rev.

[26] Glowinski J, Iversen LL (1996). Regional studies of catecholamines in the rat brain. I. The disposition of [3H]norepinephrine, [3H]dopamine and [3H]dopa in various regions of the brain. J. Neurochem.

[27] Messripour M, Haddadi H (1988). Effect of ascorbic acid administration on copper-induced changes of rat brain hypothalamic catecholamine contants. Acta. Neurol. Scand.

[28] Lowry OH, Rosebrough NJ, Farr AL, Randell RJ (1951). Protein measurement with the Folin phenol reagent. J. Biol. Chem.

[29] Devi C, Reddy G, Chetty C (2005). Developmental lead exposure alters mitochondria monoamine oxidase and synaptosomal catecholamine levels in rat brain. Int. Dev. Neurosci.

[30] Smith MP, Cass WA (2007). Oxidative stress and dopamine depletion in an intrastriatal 6-hydroxydopamine model of Parkinson›s disease. Neuroscience.

[31] Nehru B, Sidhu P (2002). Neurotoxic effects of differential doses of lead on rat brain followed by recovery. J. Trace Elem. Exp. Med.

[32] Widzowski DV, Cory-Slechta DA (1994). Homogeneity of regional brain lead concentrations. Neurotoxicology.

[33] Qian Y, Mikeska G, Harris ED, Bratton GR, Tiffany-Castiglioni E (1999). Effect of lead exposure and accumulation on copper homeostasis in cultured C6 rat glioma cells. Toxicol. Appl. Pharmacol.

[34] Prohaska JR, Smith TL (1982). Effect of dietary or genetic copper deficiency on brain catecholamines, trace metals and enzymes in mice and rats. J. Nutr.

[35] Tainer JA, Getzoff ED, Richardson JS, Richardson DC (1983). Structure and mechanism of copper, zinc superoxide dismutase. Nature.

[36] Ledig M, Fried R, Ziessel M, Mandel P (1982). Regional distribution of superoxide dismutase in rat brain during postnatal development. Brain Res.

[37] Benetti F, Ventura M, Salmini B, Ceola S, Carbonera D, Mammi S, Zitolo A, D›Angelo P, Urso E, Maffia M, Salvato B, Spisni E (2010). Cuprizone neurotoxicity, copper deficiency and neurodegeneration. Neuro. Toxicology.

[38] Ulvi H, Yigiter R, Yoldas T, Dolu Y, Var A, Müngen B (2002). Magnesium, zinc, copper contents in hair and their serum concentrations in patients with epilepsy. East J. Med.

[39] Lech T (2002). Lead, copper, zinc, and magnesium content in hair of children and young people with some neurological diseases. Biol. Trace Elem. Res.

[40] Moshtaghie AA, Ani M, Aghadavod E, Fazilati M (2007). Protective effects of selenium and zinc on changes in catecholamine levels of brain regions in lead intoxified rat. Pak. J. Biol. Sci.

[41] Sumi-Ichinose C, Urano F, Kuroda R, Ohye T, Kojima M, Tazawa M, Shiraishi H, Hagino Y, Nagatsu T, Nomura T, Ichinose H (2001). Catecholamines and serotonin are differently regulated by tetrahydrobiopterin. A study from 6-pyruvoyltetrahydropterin synthase knockout mice. J. Biol. Chem.

[42] Frieling H, Hillemacher T, Ziegenbein M, Neundörfer B, Bleich S (2007). Treating dopamimetic psychosis in Parkinson’s disease: structured review and metaanalysis. Eur. Neuropsychopharmacol.

[43] Singh N, Pillay V, Choonara Y E (2007). Advances in the treatment of Parkinson’s disease. Prog. Neurobiol.

[44] Xu SZ, Bullock L, Shan CJ, Cornelius K, Rajanna B (2005). PKC isoforms were reduced by lead in the developing rat brain. Int. J. Dev. Neurosci.

